# Characterization of the chloroplast genome and its inference on the phylogenetic position of *Incarvillea sinensis* Lam. (Bignoniaceae)

**DOI:** 10.1080/23802359.2020.1860722

**Published:** 2021-01-27

**Authors:** Xiayu Wu, Hongzhe Li, Shaotian Chen

**Affiliations:** a School of Life Sciences, Yunnan Normal University, Kunming, China; b College of Pharmaceutical Science, Yunnan University of Chinese Medicine, Kunming, China

**Keywords:** Chloroplast genome, *Incarvillea sinensis*, type, wide distribution

## Abstract

*Incarvillea sinensis* Lam. is the type of the genus *Incarvillea* Juss., and it is widely distributed, relative to other members of the genus. In this paper, we sequenced, assembled and annotated the chloroplast genome of *Incarvillea sinensis*. The complete chloroplast genome is 162,088 bps in size, with overall GC content of 39.4%. We annotated 113 unique genes in the plastome sequence, including 79 protein coding genes, 30 tRNA genes, and four rRNA genes. The phylogenetic analysis based on chloroplast genome sequences resulted in a different resolution on the relationships among subgenera from the former.

*Incarvillea* Juss. is a herb genus of the family Bignoniaceae, and it is subdivided into five subgenera, *Niedzwedzkia*, *Olgaea*, *Amphicome*, *Incarvillea*, and *Pteroscleris*, and three of them, *Amphicome*, *Incarvillea*, and *Pteroscleris*, are distributed in China (Grierson 1961; Chen et al. 2006). *Incarvillea sinensis* Lam. is the type of the genus and belongs to the subgenus *Incarvillea*, and it is widely distributed from Southwest China to Far East Russia (Chen et al. 2012). To infer the phylogenetic position of *I. sinensis*, we assembled and characterized its chloroplast genome and phylogenetically compared it with other two species from other two subgenera, *Amphicome* and *Pteroscleris*.

We collected material from Yanjin, Tibet (98°45′32.7000E, 28°36′11.7000N), and deposited the voucher (Chen1169) in the Museum of Ethnic Medicine, Yunnan University of Chinese Medicine. The total DNA was extracted from leaves using a modified CTAB method (Doyle and Doyle 1987). We amplified the plastome genome using nine universal primer pairs following the protocol of Yang et al. (2014). Purified amplification products were fragmented for the short-insert (300–500 bp) library construction according to the manufacturer’s manual (Illumina, San Diego, CA), and then sequenced by the Illumina Hiseq 2000. We deposited the clean sequencing data (6.2 Gb) in National Genomics Data Center (https://bigd.big.ac.cn/gsa/, the accession number CRA003439). Referring to the plastome of *Tecomaria capensis* (Thunb.) Spach (accession no.: MG831880.1), we assembled the complete chloroplast genome of *I. sinensis* using Geneious 8.1 (Kearse et al. 2012), and obtained a typical quadripartite molecule with the length of 162,088 base pairs (bps) and overall GC content of 39.4%, which can be recognized as the large single copy (LSC), the short single copy (SSC), and two inverted regions (IRs). The LSC, SSC, and IR regions are 82,632 bps, 8666 bps, and 35,394 bps in length, and respectively, their GC contents are 38.0%, 33.2%, and 41.6%. Finally, we annotated the molecule using Geneious 8.1 (Kearse et al. 2012), and results showed that the plastome was composed of 113 unique genes, including 79 protein coding genes, 30 tRNA genes, and four rRNA genes. We released the chloroplast genome and submitted it to GenBank (accession no.: MT937254).

To infer the position of the genus *Incarvillea* and the relationship among three subgenera, we constructed a phylogenetic tree based on the chloroplast genome sequence data from 12 species of Bignoniaceae and one outgroup from Scrophulariaceae. We aligned all 13 chloroplast genome sequences using Geneious 8.1 (Kearse et al. 2012), and determined the best-fitting model for the chloroplast genome data matrix using Modeltest v3.7 (Posada and Crandall 1998), and then maximum-likelihood analysis was performed using RAxML (Stamatakis 2014) with 1000 bootstrap replicates. The result showed that three species of *Incarvillea* comprised a clad, which was sister to *Tecomaria* Spach, while *I. sinensis* was affinitive to *Incarvillea arguta* (Royle) Royle and their clade was sister to *Incarvillea compacta* Maxim (Figure 1). This result is different from that based on ITS and trnL-F (Chen et al. 2005), which revealed that *I. arguta* was sister to the clade of *I. sinensis* and *I. compacta*. It seems that the inconsistency is caused by lots of indels in the alignment of chloroplast genome sequences.

**Figure 1.**
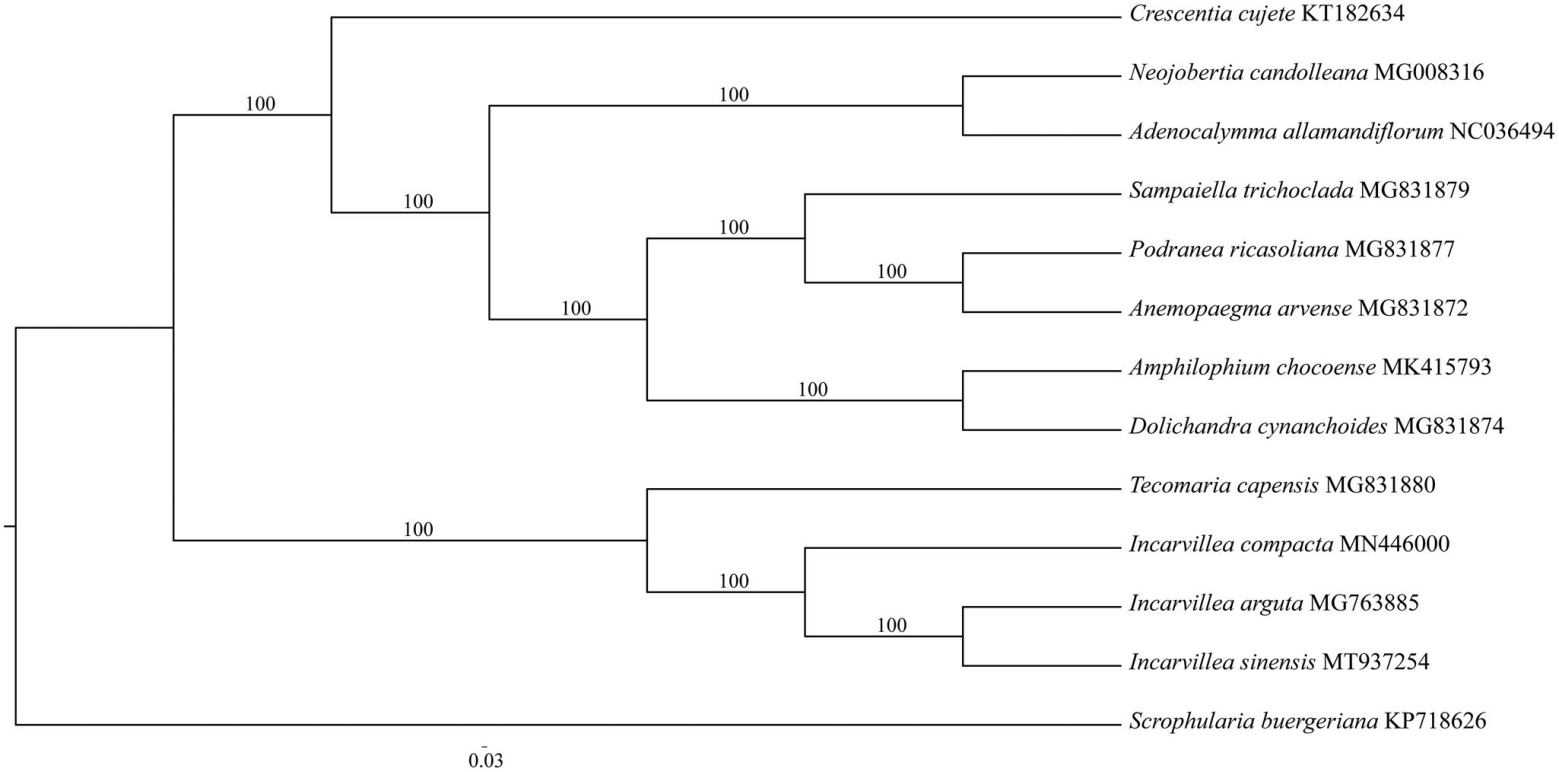
Phylogenetic position of *Incarvillea sinensis* inferred from 13 chloroplast genomes. Bootstrap support is indicated for each node.

## Data Availability

The genome sequence data that support the findings of this study are openly available in GenBank of NCBI at https://www.ncbi.nlm.nih.gov/ under the accession no. MT937254. The associated BioProject, SRA, and Bio-Sample numbers in National Genomics Data Center (https://bigd.big.ac.cn/gsa/) are PRJCA003773, CRA003439, and SAMC254838, respectively.
